# Alterations in Task-Related Brain Activation in Children, Adolescents and Young Adults at Familial High-Risk for Schizophrenia or Bipolar Disorder - A Systematic Review

**DOI:** 10.3389/fpsyt.2020.00632

**Published:** 2020-07-10

**Authors:** Line Korsgaard Johnsen, Anna Hester Ver Loren van Themaat, Kit Melissa Larsen, Birgitte Klee Burton, William Frans Christiaan Baaré, Kathrine Skak Madsen, Merete Nordentoft, Hartwig Roman Siebner, Kerstin Jessica Plessen

**Affiliations:** ^1^ Child and Adolescent Mental Health Centre, Copenhagen University Hospital, Mental Health Services, Capital Region Psychiatry, Copenhagen, Denmark; ^2^ Danish Research Centre for Magnetic Resonance, Centre for Functional and Diagnostic Imaging and Research, Copenhagen University Hospital Hvidovre, Copenhagen, Denmark; ^3^ Faculty of Health and Medical Sciences, Institute for Clinical Medicine, University of Copenhagen, Copenhagen, Denmark; ^4^ Radiography, Department of Technology, University College Copenhagen, Copenhagen, Denmark; ^5^ Mental Health Centre, Research Unit, Copenhagen University Hospital, Mental Health Services, Capital Region Psychiatry, Copenhagen, Denmark; ^6^ The Lundbeck Foundation Initiative for Integrative Psychiatric Research, iPSYCH, Aarhus University, Aarhus, Denmark; ^7^ Department of Neurology, Copenhagen University Hospital Bispebjerg, Copenhagen, Denmark; ^8^ Division of Child and Adolescent Psychiatry, Department of Psychiatry, The University Hospital of Lausanne (CHUV), Lausanne, Switzerland

**Keywords:** fMRI—functional magnetic resonance imaging, neurocognitive function, familial high-risk, schizophrenia, bipolar disorder, children, adolescents

## Abstract

Children, adolescents, and young adults with at least one first-degree relative [familial high-risk (FHR)] with either schizophrenia (SZ) or bipolar disorder (BD) have a one-in-two risk of developing a psychiatric disorder. Here, we review functional magnetic resonance imaging (fMRI) studies which examined task-related brain activity in young individuals with FHR-SZ and FHR-BD. A systematic search identified all published task-related fMRI studies in children, adolescents, and young adults below an age of 27 years with a first-degree relative with SZ or BD, but without manifest psychotic or affective spectrum disorder themselves. The search identified 19 cross-sectional fMRI studies covering four main cognitive domains: 1) working memory (n = 3), 2) cognitive control (n = 4), 3) reward processing (n = 3), and 4) emotion processing (n = 9). Thirteen studies included FHR-BD, five studies included FHR-SZ, and one study included a pooled FHR group. In general, task performance did not differ between the respective FHR groups and healthy controls, but 18 out of the 19 fMRI studies revealed regional alterations in task-related activation. Brain regions showing group differences in peak activation were regions associated with the respective task domain and showed little overlap between FHR-SZ and FHR-BD. The low number of studies, together with the low number of subjects, and the substantial heterogeneity of employed methodological approaches within the domain of working memory, cognitive control, and reward processing impedes finite conclusions. Emotion processing was the most investigated task domain in FHR-BD. Four studies reported differences in activation of the amygdala, and two studies reported differences in activation of inferior frontal/middle gyrus. Together, these studies provide evidence for altered brain processing of emotions in children, adolescents, and young adults at FHR-BD. More studies of higher homogeneity, larger sample sizes and with a longitudinal study design are warranted to prove a shared or specific FHR-related endophenotypic brain activation in young first-degree relatives of individuals with SZ or BD, as well as to pinpoint specific alterations in brain activation during cognitive-, emotional-, and reward-related tasks.

## Introduction

Schizophrenia (SZ) and bipolar disorder (BD) are severe and highly heritable (1) mental illnesses with a substantial impact on the individuals concerned, their families, and the society. By early adulthood, the offspring of parents with severe mental illnesses, including SZ, BD, and major depressive disorder, have a one-in-three risk of developing a psychotic or major mood disorder and a one-in-two risk of developing any mental disorder (2). Heritability shows partial phenotypical specificity with largest risk ratios for SZ among offspring of parents with SZ and largest risk ratios for BD among offspring of parents with BD. Additionally, offspring of parents with SZ have a significantly increased risk of BD and offspring of parents with BD have a significantly increased risk of SZ (2). According to data extracted from Danish registries, child and adolescent offspring of parents with severe mental illness express increased incidence rates for all diagnoses of child and adolescent mental disorders compared to reference offspring of parents without severe mental illness (3).

SZ is characterized as a neurodevelopmental disorder and manifests in adolescence or early adulthood (4, 5), whereas the developmental nature of BD is less understood (6). However, genome-wide association studies of SZ and BD have shown overlapping genetic risk loci (7, 8). Approximately, two-thirds of the genetic expression profile are shared across SZ and BD (9, 10). Thus, the genetic risk profile for SZ and BD may also be shared across the offspring of parents with these severe mental illnesses. Whether these disorders also share phenotypic expression profiles, e.g., brain responses, during early stages of pathogenesis is unclear.

First-degree relatives of individuals with SZ or BD, referred to as individuals with familial high-risk (FHR), show impairments on a variety of neurocognitive and motor functions on a group level, even at young age (11–17). Moreover, deficits in neurocognitive functioning in individuals with the manifest disorder and in adult FHR individuals have been linked to altered brain functioning and have been suggested as endophenotypic of the disorders (18–22). This, in turn, may reflect an increasing dysfunction during brain maturation in critical brain regions. Various childhood mental disorders including ADHD, autism, and childhood onset schizophrenia (COS) are associated with abnormal developmental trajectories for cortical thickness (23). Interestingly, healthy siblings of patients with COS show significant reductions in regional gray and white matter volume, suggesting a trait marker (24, 25). In keeping with this, characteristics of cortical morphology in child and adolescent offspring of SZ patients show cross-sectional decrease in global and parieto-occipital surface area compared to a control group, and a decrease in occipital surface area compared to offspring of BD patients (26). In that study, global and parietal surface area scaled with the expression of positive and negative prodromal symptoms in offsprings of SZ patients (26).

Throughout post-natal development, the brain undergoes continuous maturation (27–29). This is also the case for the frontal cortex, (30), but the maturational trajectories of frontal cortical areas differ from the trajectories of other inter-connected brain regions, such as the basal ganglia (31, 32). This discrepancy may render the brain vulnerable and favor the emergence of mental disorders during adolescence and early adulthood (32, 33).

Studies on the brain development in young individuals at high risk of severe mental disorders may not only reveal underlying neurobiological mechanisms but also identify markers associated with risk and resilience. Multiple studies of FHR individuals have investigated cognitive, motor or social capabilities in vulnerable populations (2, 11, 13, 17) also in combination with neuroimaging methods (34, 35), which has resulted in several systematic reviews and meta-analyses (18, 19, 36, 37). No systematic review or meta-analysis to date, however, has exclusively focused on neuroimaging studies in children, adolescents, and young adults at FHR of severe mental disorders. This is surprising, because the inclusion of adult individuals with FHR substantially impacts the interpretation of the results, as these individuals may already have passed the peak onset period and alterations may, thus, rather represent factors of resilience or compensation than vulnerability and risk.

Here, we systematically reviewed the existing literature investigating children, adolescents, and young adults at FHR for SZ (FHR-SZ) or FHR for BD (FHR-BD) with task-related functional magnetic resonance imaging (fMRI). We only considered studies of FHR individuals who had not yet manifested signs of serious mental illness. Half of all lifetime mental disorders start by the mid-teenage years and three quarters by the mid-20s (38). Therefore, we narrowed our search to studies including individuals with a group mean age under or equal to 21 years and did not include studies with participants above the age of 27 years. We aimed to answer the following questions; Firstly, do brain activation patterns associated with task-related activity in children, adolescents, and young adults at FHR-SZ or FHR-BD, differ from the patterns observed in healthy controls (HC)? Secondly, do the two FHR groups show shared or specific differences in brain activation patterns? Lastly, we relate the findings to earlier reports in patients with the manifest disorders, as well as adult FHR populations and clinical high-risk populations. Our approach aims at identifying early neurobiological alterations associated with FHR for severe mental disorders with fMRI, knowledge that may assist the optimization of diagnostic tools and improve the design of early interventions and preventive measures. Neurobiological approaches of child, adolescent and young adult offspring of patients with severe mental illnesses offer the opportunity to assess the early neural imprint of a genetic vulnerability to disease and make way for the study of preclinical features and the interaction between illness-related progressions and normal brain maturation.

## Methods

**Figure 1:**
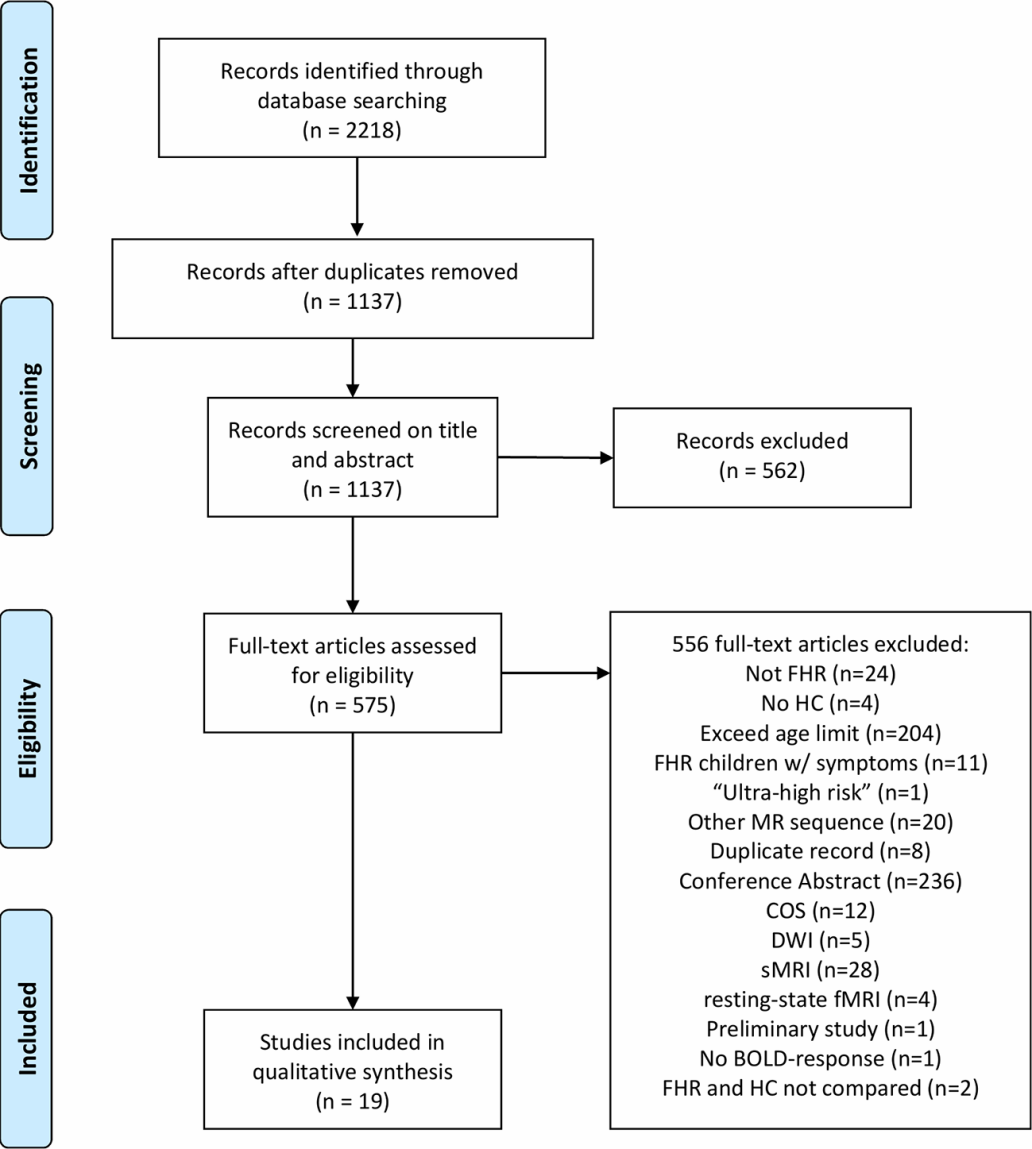
The Preferred Reporting Items for Systematic Reviews and Meta-analyses (Prisma) flow-chart illustrating the systematic review procedure. FHR, familial high-risk; HC, healthy control; MR, magnetic resonance; COS, childhood-onset schizophrenia; DWI, Diffusion-weighted imaging; sMRI, Structural magnetic resonance imaging (MRI); fMRI, functional MRI; BOLD, Blood-oxygen-level-dependent.

We combined the search terms “MRI”, “fMRI”, “children”, “adolescents”, “bipolar disorder”, “schizophrenia”, “risk”, “first-degree relative”, and “genetic predisposition” in PubMed, EMBASE, and PsychINFO followed by a screening of inclusion criteria to identify fMRI studies including children and adolescents at FHR-SZ or FHR-BD (**Table 1**). The time-period covered in our search included all publications published in this area until September 10th, 2019. The protocol for this systematic review was registered on Prospective Register for Systematic Reviews (PROSPERO; registration ID: CRD42018086995) and is available on their International website (https://www.crd.york.ac.uk/prospero/display_record.php?ID=CRD42018086995). Identification, screening, eligibility, and inclusion procedures followed the Preferred Reporting Items for Systematic Reviews and Meta-analyses (Prisma) 2009 flow diagram (39) (**Figure 1**). We screened and included articles based on the following inclusion criteria: 1) peer-reviewed articles in English; 2) study population of children, adolescents and/or young adults at FHR (familial high-risk defined as having a first-degree relative diagnosed with SZ or BD) of developing SZ or BD; 3) absence of a diagnosis in the spectrum of psychotic or affective disorders as well as no reported symptoms in these areas; 4) mean age of study population below or equal to 21 years, with no individuals above 27 years of age; 5) direct comparison with a HC group; and 6) employment of task-related fMRI. Screening, eligibility and study selection procedures were completed according to the inclusion criteria by authors AV and LJ, independently. These procedures were completed by following EndNote specific review procedures (40). Any disagreement was followed up by KP. Data extraction was done by LJ under supervision from KL and HS. Summary measures include differences in mean values for behavioral variables as well as peak activation differences between the respective FHR group and HC.

**Table 1:** Detailed overview of the three database-specific search strings applied in PubMed, EMBASE, and PsychINFO, respectively.

PubMed	Methods	Age group	Clinical	Descriptive
TX	MRI, fMRI, Neuroimaging	Children, Child, Adolescent*, Youth*	Bipolar, Schizophrenia*	Risk, “impaired parent”, “impaired parents”, “disabled parent”, “disabled parents”
MeSH term	Neuroimaging, Magnetic Resonance Imaging	Child, Adolescent	Bipolar and related disorders, Schizophrenia Spectrum and Other Psychotic Disorders	Genetic predisposition to disease, Child of impaired parents, Risk
**EMBASE**	**Methods**	**Age group**	**Clinical**	**Descriptive**
Keyword	MRI, fMRI, Neuroimaging, “Nuclear Magnetic Resonance Imaging”	Children, Child, Adolescent*, Youth*	Bipolar, Schizophrenia*	Risk, first degree relative, genetic predisposition to disease
Subject heading	Neuroimaging, Nuclear Magnetic Resonance Imaging	Juvenile	Bipolar disorders, Schizophrenia spectrum disorder	Risk, first degree relative, genetic predisposition
**PsycINFO**	**Methods**	**Age group**	**Clinical**	**Descriptive**
TX	MRI, fMRI, Neuroimaging, Magnetic Resonance Imaging”	Child, children, adolescent*, teenage*, young adults, youth	Bipolar, Schizophrenia*, schizophrenia,	Risk, genetic predisposition, “impaired parent”, “impaired parents”, “disabled parent”, “disabled parents”, first degree relative
MeSH term	Neuroimaging, Magnetic Resonance Imaging	Children, adolescent*, teenagers, young adults, youth	Bipolar disorder, bipolar, Schizophrenia, Schizophrenia disorders	Risk, genetic predisposition to disease, child of impaired parents, first degree relative

Methods, age group, clinical, and descriptive word categories was assembled by the AND function, while TX (free text)/Keyword and MeSH (Medical Subject Headings) term/Subject heading was assembled by the OR function. The asterisk (*) serves as the truncation (or wildcard) operator. Words match if they begin with the word preceding the * operator.

## Results

A total of 1,137 articles were screened by title and abstract according to the inclusion criteria listed in the *Methods* section, resulting in exclusion of 562 articles. During the eligibility procedure, we assessed 575 articles by full-text reading of which 556 full-text articles were excluded (**Figure 1**). As a result of the exclusion process, 19 task-related fMRI articles were included of which five studies included individuals at FHR-SZ, 13 studies included individuals at FHR-BD, and one study pooled FHR-SZ and FHR-BD into one FHR sample (**Table 2**). All studies employed blood oxygen level–dependent (BOLD) fMRI and identified task-related changes in regional activity at the voxel level by specifying a general linear model (GLM). Three studies performed additional psycho-physiologic interaction (PPI) analysis (41, 42, 49) and a single study applied dynamic causal modeling (53). This review only concerns task-related brain activation as revealed by a GLM based approach. We divided the included articles into four main task domains: 1) Working memory (WM), 2) Cognitive control, 3) Reward processing, and 4) Emotion processing. Note that two articles have been included in two different task domains, i.e., Ladouceur, Diwadkar (55) in WM and emotion processing, and Hart, Bizzell (54) in cognitive control and emotion processing, as the applied tasks contrasts expand both domains. Division of studies into these four main task domains was based on the empirical evidence, when regarding the study specific contrasts applied in the respective GLM designs. We employed this pragmatic and bottom-up approach when reviewing the finally included papers. For details about the study specific contrast of interest see **Supplementary Tables S1–S4**. Due to the limited total number of articles identified at this point, we included all articles concerning task-related fMRI findings in children, adolescents, and young adults at FHR-SZ or FHR-BD independent of behavioral differences between groups. We have, however, explicitly reported the behavioral differences present in the respective studies.

**Table 2:** Overview of study characteristic of included articles in the qualitative synthesis.

Study	High-risk disorder	Task	Performance	Age range	Healthy control			Familial high-risk				
					n (m/f)	Mean age (SD)	IQ	n (m/f)	Mean age (SD)	IQ	Existing diagnoses	Relation
**Working memory**	** **											
1. Bakshi et al. (41)	SZ	N-back task	Response latency; FHR-SZ > HC	8–20	25 (17/8)	14.6 (2.80)	93.8	19 (12/7)	14.3 (3.10)	93.1	SAD, ADHD, SP	Offspring
2. Diwadkar et al. (42)	SZ	Emotional face valence n-back task	No difference	HC: 10–19 FHR: 8–19	25 (17/8)	14.6 (NR)	94.0	19 (12/7)	14.3 (NR)	93.0	SAD, ADHD	Offspring
3. Thermenos et al. (43)	BD	N-back task	No difference	13–24	10 (5/5)	17.1 (1.40)	100.9	10 (5/5)	18.4 (4.20)	106.8	NR	Mix
Total					60			48				
**Cognitive control**												
4. Diwadkar et al. (44)	SZ & BD	Continuous performance task	No difference	8–20	24 (NR)	15.4 (2.70)	93.1	22 (NR)	14.1 (3.1)	94.2	NR	Offspring
5. Deveney et al. (45)	BD	Stop signal task	No difference	NR	21 (13/8)	13.8 (2.00)	113.7	13 (6/7)	13.5 (1.80)	109.0	None	Mix
6. Kim et al. (46)	BD	The change task	No difference	8–17	21 (13/8)	13.7 (1.96)	113.7	13 (6/7)	13.9 (2.02)	107.9	None	Mix
7. Pagliaccio et al. (47)	BD	Global-local selective attention task	CV RT; FHR-BD > HC	8–25	53 (21/32)	18.7 (4.09)	115.1	29 (15/14)	14.9 (3.47)	110.7	ADHD, MDD	Mix
Total					119			77				
**Reward processing**												
8. Manelis et al. (48)	BD	Number guessing reward task	No difference	7–17	23 (12/11)	13.7 (1.80)	105.8	29 (15/14)	13.8 (2.45)	103.2	MDD, ADHD, AD, ODD, phobia, TD, ED	Offspring
9. Singh et al. (49)	BD	Monetary incentive delay task	No difference	8–15	25 (10/15)	11.8 (2.37)	115.1	20 (7/13)	12.7 (2.85)	111.3	None	Offspring
10. Soehner et al. (50)	BD	Card-number guessing game	NR	9–17	21 (10/11)	14.0 (2.24)	106.1	25 (14/11)	14.2 (2.25)	100.4	DD, AD, ADHD, disruptive behavior, ED	Offspring
Total					69			64				
**Emotion processing**												
11. Brotman et al. (51)	BD	Facial emotion processing paradigm	No difference	8–19	29 (16/13)	14.9 (1.90)	110.0	15 (9/6)	14.5 (2.20)	108.2	ADHD, AD	Mix
12. Barbour et al. (52)	SZ	Continuous affective task	No difference	HC: 10–19 FHR: 8–19	25 (17/8)	14.9 (2.80)	93.3	19 (12/7)	14.3 (3.19)	93.8	SAD, ADHD, SP	Offspring
13. Diwadkar et al. (53)	SZ	Continuous visual memory task of faces with affective valence	No difference	8–20	24 (16/8)	14.6 (2.60)	92	19 (12/7)	14.3 (3.10)	96.2	SAD, ADHD, SP	Offspring
14. Hart et al. (54)	SZ	Emotional odd ball task	No difference	9–18	21 (11/10)	14.1 (2.57)	NR	21 (10/11)	14.4 (2.56)	NR	ADHD, learning disorder, AD	Mix
15. Ladouceur et al. (55)	BD	N-back task with emotional distractors	No difference	8–17	15 (4/11)	13.8 (2.70)	NR	16 (9/7)	14.2 (2.30)	NR	None	Offspring
16. Manelis et al. (56)	BD	Facial emotion processing paradigm	No difference	7–17	23 (12/11)	13.7 (1.80)	105.8	29 (15/14)	13.8 (2.45)	103.2	MDD, ADHD, AD, ODD, phobias, TD, OCD, ED	Offspring
17. Olsavsky et al. (57)	BD	Face-emotion labelling task	No difference	8–18	56 (26/30)	14.0 (2.60)	112.0	13 (7/6)	14.0 (2.40)	113.0	AD, ADHD	Mix
18. Tseng et al. (58)	BD	Face-emotion memory task	No difference	9–19	37 (16/21)	14.7 (2.29)	108.9	13 (8/5)	13.7 (2.28)	112.9	AD, ADHD	Mix
19. Welge et al. (59)	BD	Emotional visual oddball task	No difference	10–20	32 (11/21)	14.6 (3.00)	101.0	32 (9/23)	15.3 (3.00)	104.1	ADHD	Offspring
Total					262			177				

Note that Hart et al. (54) is also included in Cognitive Control and Ladouceur et al. (55) in Working Memory in the main text. FHR, Familial high-risk; HC, Healthy control; CV, Coefficient of variation; RT, Reaction time; SZ, Schizophrenia; BD, Bipolar disorder; Mix, Group was offspring and siblings combined; SD, Standard deviation; NR, Not reported; TD, Tourette’s disease; ODD, Oppositional defiant disorder; ADHD, Attention-deficit/hyper-activity disorder; SAD, Separation anxiety disorder; SP, Social phobia; AD, Anxiety disorder; MDD, Major depressive disorder; ED, Eating disorder; OCD, Obsessive compulsive disorder.

In addition to the low number of existing articles within this specific field of research, we observed a high heterogeneity in terms of behavioral tasks, analytic methods, and reporting practices within the four main task domains. This heterogeneity precluded the use of quantitative meta-analytic methods, such as activation likelihood estimation (ALE) (60).

### Task-Related fMRI Findings

#### Working Memory

**Figure 2:**
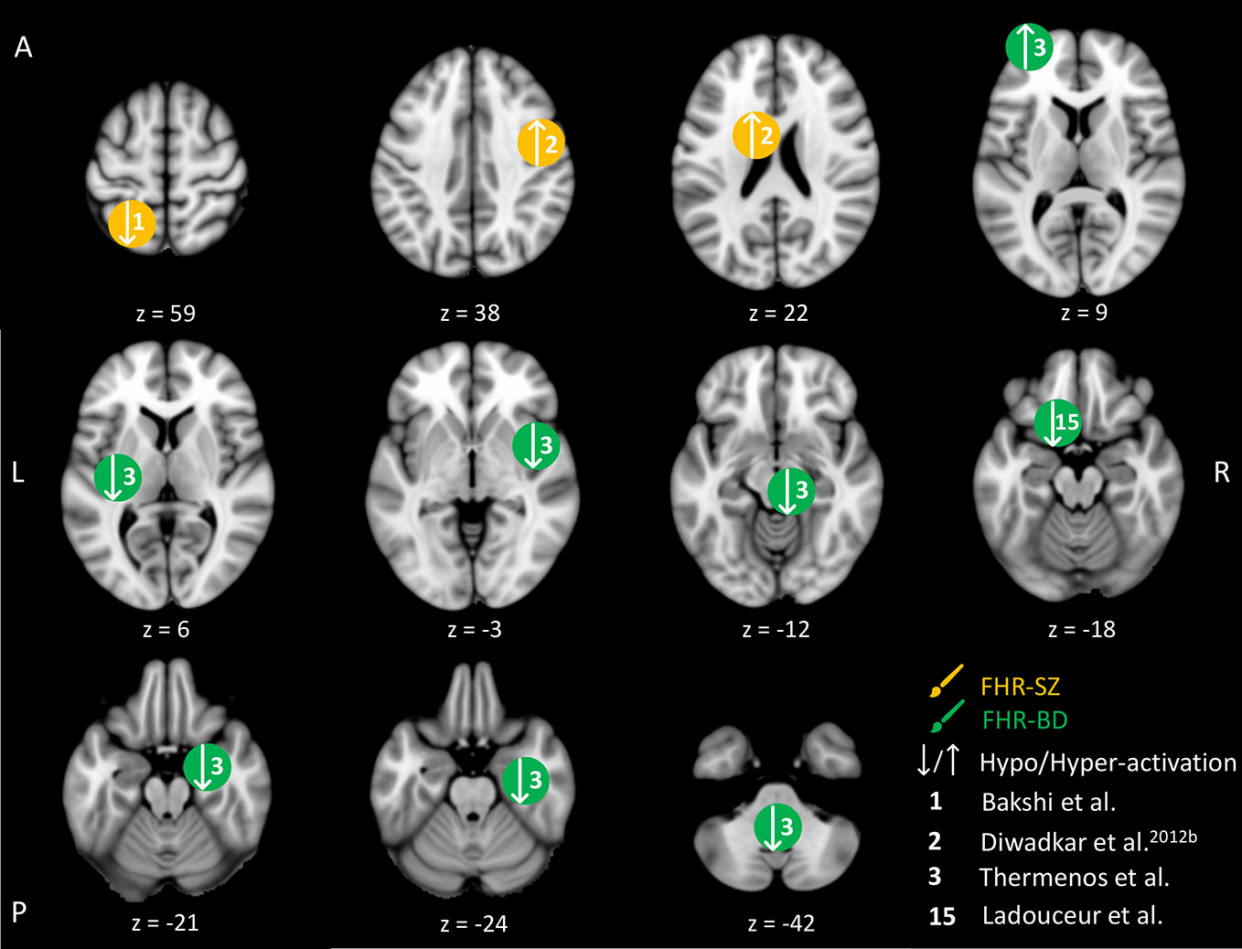
Working memory-related brain activity differences between children and adolescents at familial high-risk (FHR) for schizophrenia (SZ) and healthy control (HC, yellow circles), and for bipolar disorder (BD) and healthy control (green circles). Circles mark the peak coordinate activation difference but do not reflect the extend of activation. The coordinates of peak activation reported in Talairach space were translated to Montreal Neurologic Institute (MNI) coordinates using the MNI<->Talairach with Brodmann Areas (1.09) website (http://sprout022.sprout.yale.edu/mni2tal/mni2tal.html). Using FSLview (version 4.0.1 ^©^2004-2009 Oxford University), the reported peak coordinates in each article were entered to localize brain area on a standard MNI152 brain. For visualization of areas of peak activation differences between FHR-SZ, FHR-BD, and HC, we created figures using FSLview and Mango (Version: 4.1, Jack L. Lancaster, Ph.D., Michael J. Martinez ^©^ 2019 Research Imaging Institute, UTHS CSA). Exact coordinates and details on contrasts and statistics can be found in **Supplementary Table 1**.

Four studies were identified in young individuals at FHR-SZ or FHR-BD within the WM domain. An overview of the brain peak activity differences between individuals at FHR-SZ or FHR-BD relative to HC within the WM domain are shown in **Figure 2**. Two studies investigated individuals at FHR-SZ. In the first study, the authors found hypo-activation in the left parietal cortex in 19 FHR-SZ offspring (mean age: 14.3) relative to 25 HC (mean age: 14.6) during high versus low WM demand (**Table 2** and **Figure 2**). Furthermore, FHR-SZ relative to HC displayed lower response latencies but did not differ from HC with respect to hit rate and the effect of WM load on performance (41). In a second study, during correct versus incorrect memory performance in a WM task, it was found that 19 FHR-SZ offspring (mean age: 14.3) showed hyper-activation in the right dorsal prefrontal cortex (PFC) and left head of caudate relative to 25 HC (mean age: 14.6) (42) (**Table 2** and **Figure 2**). Of the two studies in FHR-BD, one study reported hypo-activation during high versus low WM load in 10 individuals (mean age: 18.4) in the left cerebellum, bilateral insula, as well as in the right brainstem and right para-hippocampal gyrus/amygdala relative to 10 HC (mean age: 17.1). The same task contrast in this study also elicited hyper-activation in FHR-BD relative to HC in the left frontopolar cortex (43) (**Table 2** and **Figure 2**). Another study also reported hypo-activation during high versus low WM load, though in a different area, namely the left ventro-lateral prefrontal cortex (VLPFC) in 16 FHR-BD offspring (mean age: 14.2) compared with 15 HC (mean age: 13.8) (55). None of the studies in FHR-BD summarized above found behavioral differences relative to HC.

In summary, no behavioral differences were found between FHR-BD and HC, whereas one study in FHR-SZ reported differences between FHR-SZ and HC on response latency with FHR-SZ being faster (41). Brain activity during WM performance differed between both FHR groups and HC groups in brain regions normally associated with WM processing. Although three out of four studies applied a similar high WM load versus low WM load contrast (41, 43, 55), no common altered brain activity patterns between FHR and HC were apparent between these studies. Likewise, no commonalities between FHR-SZ and FHR-BD were seen. However, without taking directionality or magnitude of the observed brain activity differences into account, the three studies, two in FHR-BD individuals and one in FHR-SZ individuals, reported altered frontal activity compared to HC. More specifically, differences were present in the left VLPFC (55), the left frontopolar cortex (43) and the right dorsal PFC (42).

#### Cognitive Control

**Figure 3:**
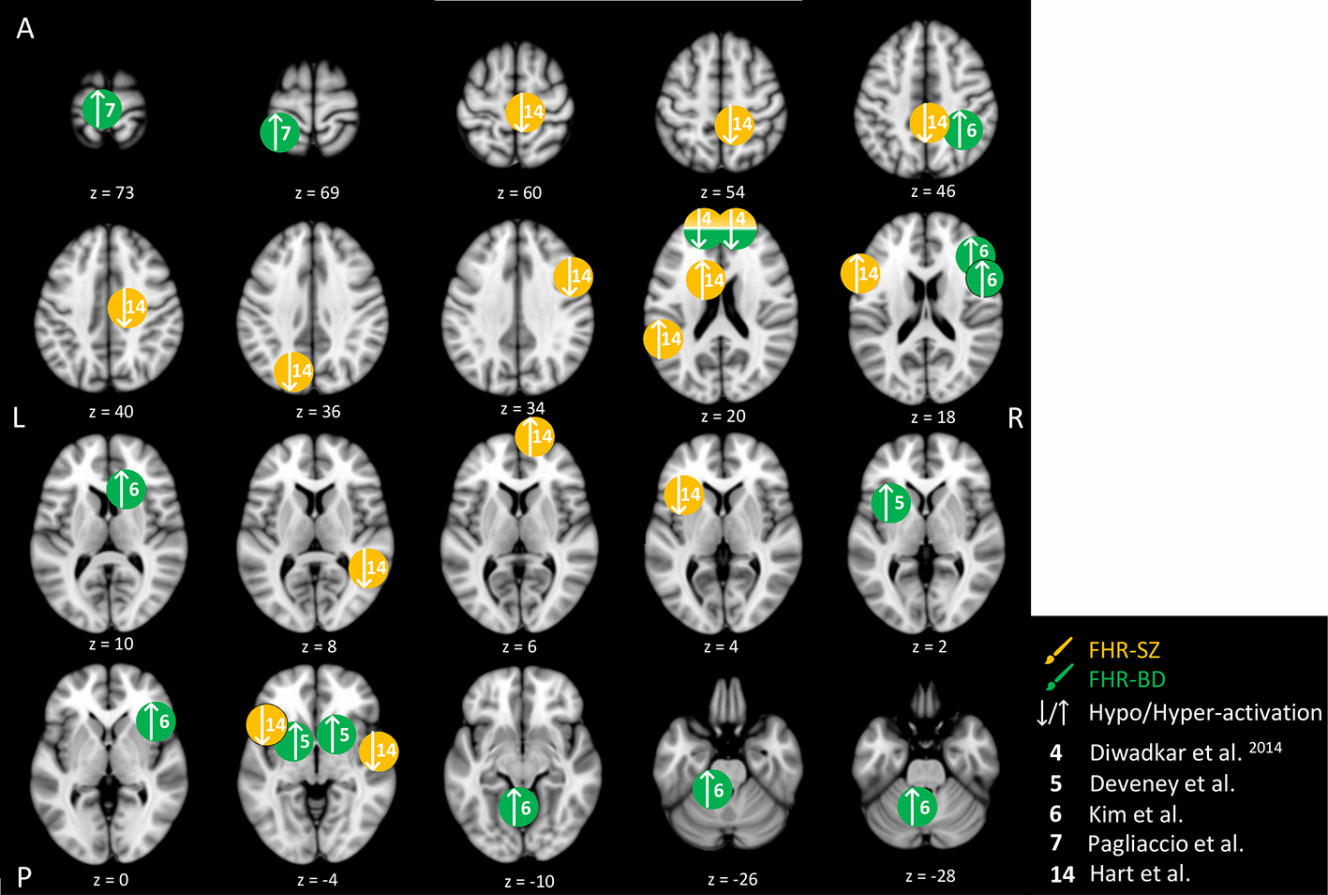
Cognitive control-related brain activity differences between children and adolescents at familial high-risk (FHR) for schizophrenia (SZ) and healthy control (yellow circles), and for bipolar disorder (BD) and healthy control (HC, green circles). Circles mark the peak coordinate activation difference but do not reflect the extend of activation. The coordinates of peak activation reported in Talairach space were translated to Montreal Neurologic Institute (MNI) coordinates using the MNI<->Talairach with Brodmann Areas (1.09) website (http://sprout022.sprout.yale.edu/mni2tal/mni2tal.html). Using FSLview (version 4.0.1 ^©^2004-2009 Oxford University), the reported peak coordinates in each article were entered to localize brain area on a standard MNI152 brain. For visualization of areas of peak activation differences between FHR-SZ, FHR-BD, and HC, we created figures using FSLview and Mango (Version: 4.1, Jack L. Lancaster, Ph.D., Michael J. Martinez ^©^ 2019 Research Imaging Institute, UTHS CSA). Exact coordinates and details on contrasts and statistics can be found in **Supplementary Table 2**.

The five included studies of FHR within the cognitive control domain have focused on attention, response inhibition, and cognitive flexibility (**Table 2**). Differences in peak brain activation in FHR-SZ and FHR-BD relative to HC within the cognitive control domain are depicted in **Figure 3**. One study pooled FHR-SZ and FHR-BD offspring into one high-genetic-risk group (n = 22, mean age: 14.1). During both high and low attention load versus passive viewing, respectively, the FHR group showed hypo-activation in the dorsal PFC and hyper-activation in the parietal cortex relative to 24 HC (mean age: 15.4). Moreover, the FHR group showed a lack of additional activation during higher attentional demand as opposed to HC (44). A second study investigated selective attention in 21 individuals at FHR-SZ (mean age: 14.4) relative to 21 HC (mean age: 14.1) with FHR-SZ individuals showing lower accuracy on identification of non-targets. In terms of brain activation, FHR-SZ relative to HC displayed hypo-activation during target identification versus task-irrelevant stimuli in the right middle frontal gyrus, left frontal operculum cortex, right supplementary motor area, left insula, right precentral gyrus, right postcentral gyrus, right superior temporal gyrus, right precuneus, and left occipital cortex. Hyper-activation during the same contrast was found in the left inferior frontal cortex, bilateral caudate, left inferior temporal gyrus, and bilateral frontal pole in FHR-SZ relative to HC (54). In a third study, using a similar selective attention task, 29 individuals at FHR-BD (mean age: 14.9) showed larger reaction time variability and displayed hyper-activation in a group-by-reaction time interaction analysis in the left medial frontal gyrus and in the left superior frontal gyrus in comparison with 53 HC (mean age: 18.7) (47). Investigating response inhibition, one study of 13 individuals at FHR-BD (mean age: 13.5) relative to 21 HC (mean age: 13.8), showed hyper-activation during successful motor inhibition (stop incorrect versus stop correct) and unsuccessful inhibition (stop incorrect versus go) in the left and bilateral putamen, respectively (45). Lastly, in a study investigating cognitive flexibility, 13 individuals at FHR-BD (mean age: 13.9) relative to 21 HC (mean age: 13.7) showed hyper-activity in the right inferior parietal gyrus, the right inferior frontal gyrus (IFG) and in the left cerebellum in change versus go trials (i.e., successful cognitive flexibility). Unsuccessful change versus go trials, i.e., unsuccessful cognitive flexibility, evoked hyper-activation in the right caudate and left cerebellum in FHR-BD relative to HC. Successful cognitive flexibility relative to unsuccessful showed hyper-activation in FHR-BD relative to HC in the right IFG (46).

In summary, two studies found behavioral differences on selective attention, one between FHR-SZ and HC (54), the other between FHR-BD and HC (47). In both studies, the FHR group performed worse than HC. Interestingly, all studies on FHR-BD groups found hyper-activity relative to HC, independent of paradigm design and task contrast (44–47) (**Figure 3**). Finally, when comparing young individuals at FHR-SZ or FHR-BD with HC during diverse cognitive control tasks, observed differences in brain activity generally involved areas within frontal, temporal, parietal, midbrain areas, as well as cerebellar sub-areas. Of note, only one study with FHR-SZ individuals contributed to these finding (54), while in another study FHR-SZ individuals were pooled with FHR-BD individuals into one high-risk group (44).

#### Reward Processing

**Figure 4:**
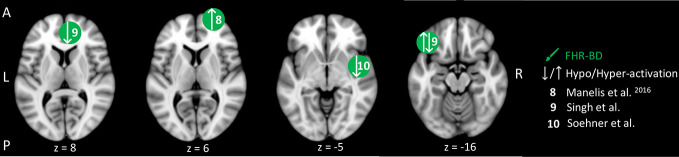
Reward-related brain activity differences between children and adolescents at familial high-risk (FHR) for schizophrenia (SZ) and healthy control (yellow circles), and for bipolar disorder (BD) and healthy control (HC, green circles). Circles mark the peak coordinate activation difference but do not reflect the extend of activation. The coordinates of peak activation reported in Talairach space were translated to Montreal Neurologic Institute (MNI) coordinates using the MNI<->Talairach with Brodmann Areas (1.09) website (http://sprout022.sprout.yale.edu/mni2tal/mni2tal.html). Using FSLview (version 4.0.1 ^©^2004-2009 Oxford University), the reported peak coordinates in each article were entered to localize brain area on a standard MNI152 brain. For visualization of areas of peak activation differences between FHR-SZ and HC, and between FHR-BD and HC, we created figures using FSLview and Mango (Version: 4.1, Jack L. Lancaster, Ph.D., Michael J. Martinez ^©^ 2019 Research Imaging Institute, UTHS CSA). Exact coordinates and details on contrasts and statistics can be found in **Supplementary Table 3**.

Three studies investigated reward processing in children, adolescents, and young adults at FHR-BD and HC (**Table 2**). Independent of winning or losing versus control trials, 29 FHR-BD offspring (mean age: 13.8) showed hyper-activation in the right frontal pole relative to 23 HC (mean age: 13.7) (48) (**Figure 4**). In another study, hyper-activation was reported in the right posterior insula in 25 FHR-BD offspring (mean age: 14.2) relative to 21 HC (mean age: 14.0) when contrasting the winning versus the control condition (50) (**Figure 4**). On the other hand, anticipation of loss versus non-loss resulted in hypo-activation in 20 FHR-BD offspring (mean age: 12.7) in the right pregenual cingulate cortex relative to 25 HC (mean age: 11.8). Furthermore, FHR-BD offspring also showed hyper-activation during reward feedback versus non-reward feedback in the left lateral orbitofrontal cortex relative to HC (49) (**Figure 4**).

In summary, differences in brain activity during reward processing in young individuals at FHR-BD relative to HC were mainly found in frontal cortical areas (**Figure 4**). Depending on the contrast of interest, i.e., anticipation or feedback, and winning or losing trials, these activation differences consisted of both hypo- and hyper-activity relative to HC. Although two studies applied similar number guessing reward tasks in FHR-BD [i.e., Manelis, Ladouceur (48) and Soehner, Bertocci (50)], activity differences were present in distinct areas and of opposite direction leaving little overlap in findings. Of note, we did not identify any studies investigating reward processing in young individuals at FHR-SZ.

#### Emotion Processing

**Figure 5:**
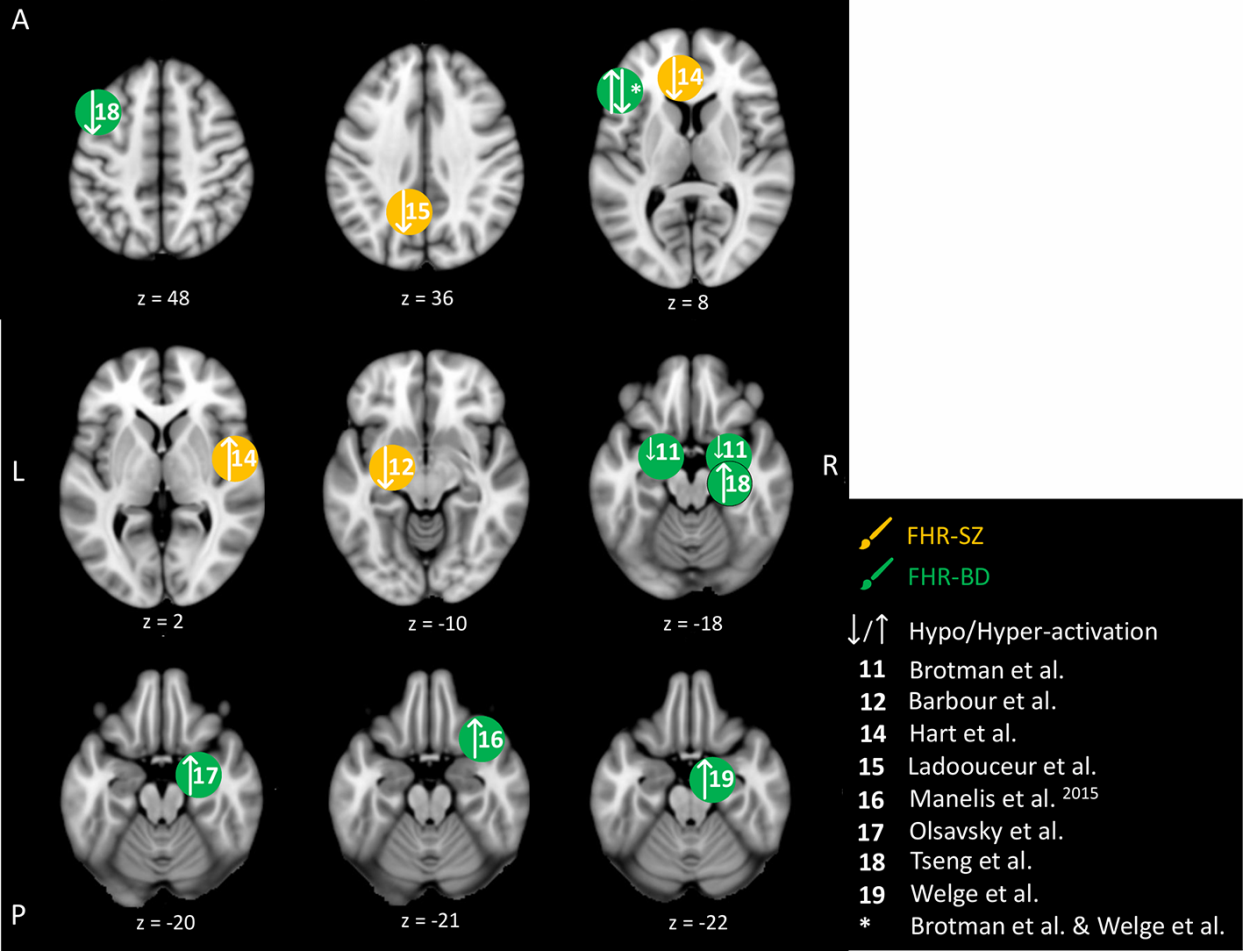
Emotion-related brain activity differences between children and adolescents at familial high-risk (FHR) for schizophrenia (SZ) and healthy control (yellow circles), and between bipolar disorder (BD) and healthy control (HC, green circles). Circles mark the peak coordinate activation difference but do not reflect the extend of activation. The coordinates of peak activation reported in Talairach space were translated to Montreal Neurologic Institute (MNI) coordinates using the MNI<->Talairach with Brodmann Areas (1.09) website (http://sprout022.sprout.yale.edu/mni2tal/mni2tal.html). Using FSLview (version 4.0.1 ^©^2004-2009 Oxford University), the reported peak coordinates in each article were entered to localize brain area on a standard MNI152 brain. For visualization of areas of peak activation differences between FHR-SZ, FHR-BD, and HC, we created figures using FSLview and Mango (Version: 4.1, Jack L. Lancaster, Ph.D., Michael J. Martinez ^©^ 2019 Research Imaging Institute, UTHS CSA). Exact coordinates and details on contrasts and statistics can be found in **Supplementary Table 4**.

We identified nine studies in young individuals at FHR-SZ or FHR-BD, dealing with brain responses related to the processing of faces showing emotional expressions or pictures with affective valence. This makes the emotion processing domain the most investigated domain in the present reviewed literature (**Table 2**). An overview of the emotion-related peak brain activity differences between young individuals at FHR-SZ and HC, and young individuals at FHR-BD and HC are depicted in **Figure 5**. None of the included studies reported behavioral differences between any FHR group and HC. One study did not find activation-related differences between 19 FHR-SZ offspring (mean age: 14.3) compared with 24 HC (mean age: 14.6) when viewing faces with emotional expressions versus distorted images (53). On the contrary, other studies employing similar emotion processing paradigms found that young FHR-BD individuals relative to HC show amygdala hyper-activation during viewing of emotional faces versus shapes (56), when viewing faces and rating them as fearful versus passive viewing (57), as well as during successful versus unsuccessful encoding of emotional faces (58). Further, distraction versus no distraction by happy faces during WM performance elicited hyper-activation in the right VLPFC in 16 FHR-BD (mean age: 14.2) relative to 15 HC (mean age: 13.8) (55). Moreover, 15 FHR-BD (mean age: 14.2) relative to 29 HC (mean age: 14.9) displayed hypo-activation in bilateral amygdala and in the left IFG with increasing anger intensity in face expressions, as well as in the left IFG during increasing happiness intensity in face expressions (51). In line with this, 19 FHR-SZ offspring (mean age: 14.3) relative to 25 HC (mean age: 14.9) showed hypo-activation in the left amygdala (centro-medial nuclei) in response to happy face expressions (52). Another study, reported hypo-activation in the left anterior cingulate cortex (ACC) and the left precuneus but hyper-activation in the central opercular cortex in 21 FHR-SZ (mean age: 14.4) relative to 21 HC (mean age: 14.1) in response to unpleasant images (53). Finally, 32 FHR-BD offspring (mean age: 15.3) relative to 32 HC (mean age: 14.6) showed hyper-activation in the left IFG during the viewing of unpleasant and pleasant images versus neutral images (59).

In summary, young individuals at FHR-BD and FHR-SZ did not show behavioral differences compared with HC in the studies investigating emotional processing included in the present review. Across emotion processing paradigms, four studies reported altered activation (i.e., hypo- or hyper-activation) of amygdala in FHR-BD individuals compared with HC (51, 56–58), although each study applied individual task contrasts. FHR-BD individuals also showed altered activation in inferior/middle FG across two different studies and task contrasts (51, 59). Findings in FHR-SZ were less clear; one study showed amygdala hyper-activation (52), while another study showed ACC hypo-activation and central opercular cortex hyper-activation (54), which may relate to differences in task setup as well as applied analytical methods.

## Discussion

We have reviewed the existing literature to clarify whether children, adolescents, and young adults at familial high risk for either schizophrenia (FHR-SZ) or bipolar disorder (FHR-BD) show altered brain activation during task performance when compared to HC, and to determine shared or distinct patterns of brain activation in FHR-SZ versus FHR-BD. In this review, we have focused on task-based functional brain alterations with a specific emphasis on the susceptibility to SZ and BD, given the significant heritability for SZ or BD. The genetic variations that have shown to confer increased life-time risk to develop SZ or BD show a large overlap (7–10). This explains why a child’s vulnerability is not exclusively associated with a high-risk to develop the same illnesses as the parent, but vulnerability applies to a broader range of psychiatric and neurodevelopmental disorders (2, 3).

The main results of this review revealed that young individuals at FHR-SZ or FHR-BD did not show behavioral impairments in the experimental setting of a neuroimaging environment. Normal task performance in fMRI studies contrasts with several neuropsychological studies that assessed the behavioral performance only (11–17). Only two fMRI studies reported performance differences in FHR-SZ in a WM-related task and in an attention-related task, respectively, and only one study found impairments of performance in FHR-BD in an attention-related task. Across all task domains, FHR-SZ and FHR-BD showed altered brain activation when compared to HC. In the absence or the presence of a minimal overlap in altered brain activation between FHR-SZ and FHR-BD, the existing fMRI data suggest that FHR-SZ and FHR-BD are associated with distinct patterns of aberrant brain activation. This evidence, however, is of circumstantial character as most studies did not directly contrast brain activation patterns between the two groups.

Within the WM domain, young individuals with FHR-SZ or FHR-BD show altered brain activation patterns in frontal cortex, including the left VLPFC and left frontopolar regions compared to HC. These findings are in line with the existing neuroimaging literature in adult individuals at FHR-SZ (61), as well as in individuals at clinical high-risk (e.g., first-episode patients) of SZ (62) and confirmed SZ and BD patients (63). WM encompasses the ability to maintain information in an easily accessible state over short periods of time to enable goal-directed behavior (64). Lesion studies, transcranial magnetic stimulation (TMS) studies, and neuroimaging studies in humans, as well as single-cell recordings in monkeys have pinpointed the DLPFC, areas in the parietal cortex (superior, ventral and inferior), the cerebellum, striatum, and the medial temporal lobe (MTL) as being the significant brain regions involved in WM processes (65). Impairment of WM is a well-known feature in individuals with SZ spectrum and has been shown to be associated with PFC and fronto-striatal dysfunction in adult individuals with SZ and their unaffected adult first-degree relatives (66). WM deficits in patients with BD are one of the most frequently observed cognitive impairments and have been linked to brain dysfunction (i.e., hyper-activation) in frontal areas (67). Similarly, WM impairments have been shown in healthy adult first-degree relatives of BD patients (15). We did not find evidence of an impaired WM performance in young individuals at FHR-SZ or FHR-BD, and the alterations in WM-related brain activation in frontal regions resembled the pattern present in adult FHR individuals (18, 36). Taken together, these findings may indicate a compensatory mechanism or reflect neural inefficiency.

In the cognitive control domain, FHR individuals showed widespread altered brain activation in areas supporting cognitive control compared to HC. However, this finding was almost exclusively obtained in FHR-BD, and the number of studies and participants producing these widespread differences in activation patterns was low. Of note, cognitive control unifies several top-down cognitive processes supporting attention, problem-solving abilities and making appropriate decisions (64). Due to the small number of studies, however, we pooled all studies related to cognitive control, although some studies were more focused on attentional demands while others tapped into inhibitory abilities. Therefore, it is not surprising that there was little to no overlap between reported results in terms of brain areas that showed peak activation differences. Decreased attention span and poor inhibitory control are well-characterized deficits associated with SZ (68), which has been linked to abnormal brain activation in the DLPFC, ACC, thalamus, and in inferior/posterior parietal areas (69). These brain areas are known to support cognitive control task performance in HC (70). Further, a study on adult FHR-SZ (mean age >21) also found abnormalities in prefrontal activation during cognitive control tasks (22). One study included in the present review investigated young individuals at FHR-SZ, showing that altered brain activation in frontal, medial and parietal brain regions associated to cognitive control performance is already expressed in young individuals at FHR-SZ (54). Similarly, symptomatic individuals with BD show impaired cognitive control, such as sustained attention and inhibitory control, which persists in remission (15, 71). During cognitive control-related task performance, altered brain activation in IFG and limbic areas have been observed in individuals with confirmed BD as well as in adult individuals at FHR-BD (mean age >21) (18, 72). In summary, the abnormal fMRI activation patterns in the cognitive domain in young individuals with FHR match the findings reported in studies of individuals with confirmed SZ or BD as well as adults at FHR-BD and adults at FHR-SZ.

Studies in the reward processing domain only investigated young individuals at FHR-BD and showed differences in brain activity in frontal and medial areas during reward processing relative to HC. Depending on the investigated contrast of interest in the different studies reflecting brain responses to, e.g., anticipation, feedback, winning or losing, these activation differences in individuals at FHR-BD consisted of hypo- and hyper-activation relative to HC. Reward is a central component for facilitating motivation-based learning and the learning of appropriate responses to stimuli, as well as the formation of habits (73). The foundation of the reward system consist of circuits connecting specific frontal- and basal ganglia regions, including the ACC, the orbitofrontal cortex, the ventral striatum, the ventral pallidum, and the midbrain regions (74, 75). Manic episodes, a core symptom in BD, have been associated with impulsive decision making and risk taking that may arise from hyper-sensitivity to reward or general reward dysregulation (76). In the absence of reward-related behavioral impairments in young individuals at FHR-BD, the altered activation patterns of reward-related brain regions observed in the present review may indicate hyper-sensitivity or dysregulation in response to reward-related cues or compensatory mechanisms. We did not identify any studies investigating reward-related brain activity in young individuals at FHR-SZ compared with HC although negative and positive symptoms in SZ may relate to dysfunction of the reward system in the brain (77).

Emotion processing was the most investigated cognitive domain in children, adolescents, and young adults at FHR-BD. Altered amygdala function in individuals at FHR-BD was reported across different emotion processing studies (51, 56–58). Also, IFG/middle FG showed altered activation in FHR-BD compared with HC across two different studies and task contrasts (51, 59). These alterations are consistent with literature investigating BD patient populations, underlining these deficiencies as possible endophenotypic markers of the disorder (78). The two studies focusing on FHR-SZ yielded less consistent results, showing hypo-activation in amygdala in response to positive faces (52), and in ACC in response to aversive images (54). Viewing of aversive images also elicited hyper-activation in the central opercular cortex in FHR-SZ compared to HC (54). The neurobiological alterations in FHR-SZ youth compared to HC is adding to a growing body of evidence for endophenotypic traits of SZ which is also present in individuals at ultra-high risk (UHR) for psychosis, showing hyper-activation of frontal areas compared to controls when viewing negative pictures (79). The ability to regulate and process emotional responses depending on affective and social cues enables appropriate adaptiveness in various events throughout the lifespan (80). Subcortical structures, such as the amygdala and other limbic areas, play a key role in the processing of emotions. Emotion dysregulation is a core feature of almost every severe mental disorder, causing maladaptive decision making and social interactions (81). Patients with SZ and BD show an affective bias toward erroneous interpretation of emotional stimuli and general emotion dysregulation (82, 83). Individuals with SZ are impaired when making affective judgment and regulation, which may lead to misinterpretation of social cues and poor social skills (84). Individuals with BD show deficits in emotional processing even during euthymic periods (85). Of note, individual studies included in the emotion processing domain in the present review spanned several behavioral paradigm designs, but individual analytical approaches incorporated an emotion processing contrast (see **Supplementary Table S4**). Despite heterogenous task setups, reported results were fairly converging on differences between FHR-BD and HC on amygdala and IFG acivation.

A between-group comparison, including confirmed SZ or BD, FHR, and HC groups, may expand the current knowledge in three directions. First of all, brain-based measures found in FHR individuals and in individuals with confirmed SZ or BD, but not in HC, may reflect neurobiological markers of risk for severe mental illness, and thus may be classified as potential risk endophenotypes (86). Second, the comparison may also identify potential biomarkers for resilience to severe mental illness (87). This may be the case for brain regions where FHR individuals showed regional increases in brain activity, relative to confirmed SZ, BD, and HC, and where task-related brain activity scales positively with task performance. Finally, regions where individuals with confirmed SZ or BD showed dysfunction relative to FHR and HC may reflect potential illness-related adaptations. In these areas, regional brain activity should not reflect high quality of performance, but may rather scale with measures of task impairment. These neurobiological illness-related adaptations may be heavily influenced by important factors, such as duration of illness, medication, illness onset time, etc. As earlier stated, attenuated symptoms in psychosis may precede the manifest disorder.

An alternative way to investigate this hypothesis is by examining whether behavioral and neurobiological impairments are already present in individuals with an At-Risk-Mental-State (ARMS), also known as individuals at UHR for psychosis. This UHR category was introduced with the goal of developing preventive strategies of psychotic disorders and requires individuals to present with either (a) positive symptoms that are typical of psychotic disorders but of subthreshold severity or duration or (b) genetic high risk accompanied by functional decline (88). Individuals at UHR have an increased risk for developing psychosis with transition rates of 29% after 2 years (89, 90). As all UHR criteria rely on help-seeking individuals, the prevalence of the UHR state in the general population is not known to date. Widespread mild cognitive deficits are present in UHR individuals, falling at a level in between that of healthy individuals and those with confirmed SZ, and the magnitude of deficits is comparable with those at FHR (91). Further, abnormalities in brain activity and/or functional connectivity during a variety of cognitive tasks, including verbal memory and WM, verbal fluency, social cognition, as well as in the context of functions directly associated to the emergence of psychotic symptoms has also been shown [for a review, see Andreou and Borgwardt (92)]. Of note and as was the results of the present review, neuroimaging abnormalities in UHR is observed even in the absence of differences in behavioral performance (92), which may point to a compensatory mechanisms for the brain circuits to uphold sufficient behavior.

Behavioral differences between FHR individuals and HC were limited to the domains of WM and cognitive control. FHR-SZ showed lower response latency during an n-back task (41), and poorer performance in detecting non-targets compared to HC during an emotional odd ball task (54). Further, individuals with FHR-BD compared with HC displayed higher reaction time variability in a global attention task (47). The lack of behavioral differences in most studies summarized in the present review may be due to the chosen behavioral variables (e.g., accuracy and reaction time) that may lack sufficient sensitivity. Previous discussions have focused on the limitations of measuring high-level cognitive processes by simply inferring on accuracy and reaction time variables, and argue that some aspects of cognitive processes may be detectable with fMRI but not with these crude behavioral variables (93). Recent computational efforts and behavioral modeling approaches such as Bayesian modeling could help disentangle, explain, and even predict the different contributions from various behavioral sub-parameters within behavioral domains and task paradigms, which may then show a behavioral separation between children, adolescents, and young adults at FHR-SZ or FHR-BD, and HC. An important factor to consider may also be the effect of specific paradigm designs to fit them into an fMRI setting. During the recording of fMRI, a collection of images covering the whole brain is generally acquired every 1–3 s, and hundreds of brain volumes are gathered during completion of an entire fMRI scan, lasting around 2–15 min (94). In this setting, the time constraints for the cognitive tasks need adjustment to this temporality. The limited temporal resolution inherent in fMRI acquisition thus narrows the complexity and speed with which the cognitive paradigm may be carried out and is different from the cognitive paradigms completed outside a neuroimaging environment.

Sample sizes in the included studies were in general small to moderate of size with the smallest being 10 and the largest being 56. The mean sample size of all included studies was 26 for HC and 20 for FHR. Large effect sizes (Cohen’s d > 0.8) have been found in individuals with manifest BD when tested on different cognitive domains, but in first-degree relatives effect sizes are generally small to medium (Cohen’s d < 0.5) yet still significantly different from HC (15). In addition, individuals with SZ perform on average about 0.92 standard deviations worse than controls across many cognitive tasks, whereas the average effect size in healthy relatives on the same metric compared with appropriate control groups is approximately 0.35 (19). Hence, patient studies generally require smaller sample sizes, whereas studies of relatives require larger sample sizes to observe statistically significant group differences. Therefore, valuable evidence may have been missed due to insufficient statistical power inherent in small sample sizes. Also, when investigating a heterogeneous group of individuals (i.e., age span, sex, FHR disorder, comorbidities, etc.) small sample sizes could further obscure significant findings. In addition to small sample sizes in included studies, overlapping of study populations may present a potential confounding factor. Several studies may have originated from the same overall study and/or shared recruitment details and methods, and may thus not stem from independent populations, however, we did not investigate this further.

To fully establish a link between task-related activity and risk or resilience factors of psychiatric disorders, longitudinal fMRI data needs to be acquired. Indeed, longitudinal studies following symptom-free at risk or vulnerable individuals through maturation and possible development of severe mental illnesses would allow for identification of relevant pre-clinical and clinical markers of vulnerability, disease onset and/or resilience. The cross-sectional design of the studies included in the present review is thus a major limitation. While cross-sectional studies allow mapping of neurobiological differences between young individuals at FHR and typically developing controls, observed group differences may reflect vulnerability, brain alterations leading to disorder, or compensational mechanisms as well as variability in developmental stage. As an example of variation, grey matter volume in the frontal cortex peaks between age 7 to 11, but total cortical volume can vary up to 50% between typically developing individuals which enhances the relevance of inter-individual variability (95). Likewise, the subcortical structures undergo developmental changes in volume and shape in a non-linear fashion (96). General for the studies included here is that they do not report other environmental factors than socio-economic status and parental educational level. Since FHR studies cannot disentangle the effects of shared genes from shared environmental influences, designing studies with focus on, e.g., epigenetic analysis as well as home-, school-, and work- environment, and adverse lifetime events might contribute to a better understanding of contributing factors in the complex development of both SZ and BD.

In line with the longitudinal perspective mentioned above, the age of the included participants, as well as the age-range in the groups are of importance when evaluating vulnerability factors. Investigating young individuals at FHR for severe mental illnesses is of importance given the brain’s susceptibility to maladaptive changes within this period of the lifespan (33). The studies reviewed herein included individuals within an age range of at least seven years [age span 8–15 (49)] and at maximum 17 years [age span 8–25 (47)] This is a significant age span when considering neurodevelopment, since both grey and white matter undergoes substantial maturation throughout childhood and adolescence, with different structures reaching adult maturity level at different time points in different individuals (97). Including individuals within a broad age span during development may therefore include unwanted variability, which subsequently may impede clear conclusions. Future studies should thus strive to establish longitudinal cohorts with a limited age span.

Inclusion of FHR individuals with comorbid diagnoses, such as ADHD, anxiety disorder, phobia, major depressive disorder, etc. (see **Table 2** for details) varied considerably across the different included studies and consequently some of the investigated individuals at FHR were on medication. Specifically, 13 of the included studies in the present review investigated FHR individuals with existing diagnoses (41, 42, 47, 48, 50, 54, 56, 59). Of importance, the reported diagnoses could be associated with behavioral and neurological changes not related directly and exclusively to the FHR status, but to the specific diagnoses or medications. For example, it has been proposed that whole-brain immature functional connections may underlie ADHD (98) and medication-naïve children with ADHD display reduced error-signaling within cingulo-opercular regions (99). However, it is also important to consider that individuals at FHR-SZ with ADHD might constitute a subgroup with enhanced risk for psychosis compared to FHR-SZ without diagnoses (100). Hence, these participants constitute an important group when studying underlying neural vulnerability factors for psychosis. Most of the studies that included FHR individuals with existing diagnoses performed exploratory or *post hoc* analyses in which participants with existing diagnoses/non-medication naïve were excluded. The *post hoc* analyses ruled out effects of diagnoses on the main results (41, 42, 47, 48, 50, 51, 54–58). Three studies did not perform such *post hoc*/exploratory analyses (52, 53, 59) and the effect of existing diagnoses on these particular results is therefore speculative.

Here, we specifically focused on reviewing studies including young individuals with a mean age ranging from 12.7 to 18.4 years of age (11.8 to 18.7 years of age for controls). Even though this is an advantage, given that this age is before the usual onset of SZ or BD, it narrowed the number of included articles substantially. Lastly, the decision to include only publications in English further reduced the number of included articles in this systematic review.

## Conclusion

Mapping functional brain alterations in offspring of parents with confirmed SZ or BD may provide important insights into the underlying neurobiological processes that convey vulnerability to these disorders. Given the heterogeneity of the measures, methods and findings, supplementary fMRI studies are needed. These studies should preferentially aim at a longitudinal design and include large groups of individuals with a narrow age range to facilitate the interpretation of altered activity patterns during a specific cognitive task. Our literature search yielded no study that directly compared task-related brain activation between young individuals at FHR-SZ and young individuals at FHR-BD and HC. Given the empirical evidence by Lichtenstein et al. (7) and Schulze et al. (8) mentioned in the introduction section of the present review, the comparison between studies investigating FHR-SZ and studies investigating FHR-BD in larger groups and with more homogeneous methods will hopefully allow for a dissociation between the early stages of pathogenesis of these severe mental illnesses in the future. Ultimately, the understanding of the neurobiological mechanisms will guide and optimize future treatment and prevention practices toward higher precision.

## Data Availability Statement

The raw data supporting the conclusions of this article will be made available by the authors, without undue reservation, to any qualified researcher.

## Author Contributions 

LJ, AV, BB, HS, and KP contributed to conception and design of the review. LJ and AV organized the database. LJ wrote the first draft of the manuscript. KL and KP wrote sections of the manuscript. All authors contributed to the article and approved the submitted version.

## Conflict of Interest

HS has received honoraria as speaker from Sanofi Genzyme, Denmark and Novartis, Denmark, as consultant from Sanofi Genzyme, Denmark and as senior editor (NeuroImage) and editor-in-chief (NeuroImage Clinical) from Elsevier Publishers, Amsterdam, The Netherlands. HS has also received royalties as book editor from Springer Publishers, Stuttgart, Germany and Gyldendahl Publishers, Copenhagen, Denmark.

The remaining authors declare that the research was conducted in the absence of any commercial or financial relationships that could be construed as a potential conflict of interest.
